# Deciphering clonality in aneuploid breast tumors using SNP array and sequencing data

**DOI:** 10.1186/s13059-014-0470-7

**Published:** 2014-10-01

**Authors:** Ingrid M Lönnstedt, Franco Caramia, Jason Li, Debora Fumagalli, Roberto Salgado, Andrew Rowan, Max Salm, Nnennaya Kanu, Peter Savas, Stuart Horswell, Stephan Gade, Sibylle Loibl, Patrick Neven, Christos Sotiriou, Charles Swanton, Sherene Loi, Terence P Speed

**Affiliations:** Bioinformatics Division, The Walter and Eliza Hall Institute of Medical Research, 1G Royal Parade, Parkville, VIC 3052 Australia; University of Melbourne, Melbourne, VIC 3010 Australia; Division of Research and Cancer Medicine, Peter MacCallum Cancer Centre, East Melbourne, VIC 3002 Australia; Breast Cancer Translational Research Laboratory, Institut Jules Bordet, Brussels, Belgium; Cancer Research UK, London Research Institute, Translational Cancer Therapeutics Laboratory, 44 Lincoln’s Inn Fields, London, WC2A 3LY UK; Bioinformatics and BioStatistics, Cancer Research UK, Lincoln’s Inn Fields, Holborn, London WC2A 3LY UK; Translational Cancer Therapeutics Laboratory, UCL Cancer Institute, Paul O’Gorman Building, University College London, 72 Huntley Street, London, WC1E 6DD UK; German Breast Group (GBG), Neu Isenburg, Germany; Multidisciplinary Breast Centre and Gynaecological Oncology, KU Leuven, University of Leuven, Department of Oncology, B-3000 Leuven, Belgium; UCL Cancer Institute, Paul O’Gorman Building, University College London, 72 Huntley Street, London, WC1E 6DD UK; Department of Mathematics and Statistics, University of Melbourne, Melbourne, VIC 3010 Australia

## Abstract

Intra-tumor heterogeneity concerns the existence of genetically different subclones within the same tumor. Single sample quantification of heterogeneity relies on precise determination of chromosomal copy numbers throughout the genome, and an assessment of whether identified mutation variant allele fractions match clonal or subclonal copy numbers. We discuss these issues using data from SNP arrays, whole exome sequencing and pathologist purity estimates on several breast cancers characterized by ERBB2 amplification. We show that chromosomal copy numbers can only be estimated from SNP array signals or sequencing depths for subclonal tumor samples with simple subclonal architectures under certain assumptions.

**Electronic supplementary material:**

The online version of this article (doi:10.1186/s13059-014-0470-7) contains supplementary material, which is available to authorized users.

## Background

Genomes can vary between cells within a tumor. Mutations and copy number (CN) alterations which appear during tumor development result in genomic subclones emerging. Subclonality of tumors is referred to as intra-tumor heterogeneity (ITH), a topic which has attracted much attention over the last few years [1–17]. The subclones within a tumor may display different features and respond differently to drugs. It has been speculated that heterogeneity-related endpoints - a tumor’s clonal architecture, features of the subclones, or whether mutations are clonal (present equally in all tumor cells) or subclonal - might serve as biomarkers for drug resistance [5,18,19].

Heterogeneity of cancers has been studied by comparing mutations and CN alterations between spatially separated [3,6,7] or sequential [10] samples from the same tumor, or between primary and secondary tumors [11] from the same patient.

To meet clinical demand, recent studies have attempted to assess heterogeneity from single tumor samples based on whole genome sequencing (WGS) [4,8,9,12,14–16] or the cheaper whole exome sequencing (WES) [1,2,5,13,17], usually in combination with genome-wide data from SNP arrays. In general, the average CN across all cells in the tumor sample is estimated at numerous genomic positions from SNP arrays or sequencing data, and these values are joined up into genome segments of constant CN (from now on called segmented CN data). Next, the variant allele fraction (VAF) of each somatic mutation identified in the sequencing data is compared to the local CN estimate, in order to classify the mutation as clonal or subclonal. Some papers proceed to construct a phylogenetic tree which visualizes the subclonal evolution of the tumor [8,9,14–17].

We have looked at 52 single samples from newly diagnosed *HER2*-positive breast cancer tumors in the RESPONSIFY project [20] using Affymetrix SNP 6.0 arrays, WES and pathologist purity estimates. Our tumors all show heterogeneity, in that most are highly aneuploid throughout most of the genome in only a fraction of the cells. The scientific question driving this methodology project was whether identified mutations are clonal or subclonal. In particular, we hoped to assess clonality of specific CN alterations, such as those of *HER2*, by inferring the status of mutations present at their genomic location. It turns out, as we will demonstrate, that classification of mutations in samples with heterogeneity is not always possible with the data we had.

The focus of this paper is on the stages of analysis preceding automatic approaches which take input data and return an estimated clonality status of each mutation. Our principal aim is to highlight challenges in CN estimation infrequently acknowledged in the literature which influence mutation classification. We also propose solutions that may aid in the quantification of ITH in tumor samples that have high levels of CN alteration. Such a method will help in understanding how ITH is related to prognosis, that is, survival of patients diagnosed with breast cancers, as well as drug resistance, as it will be applicable to large datasets with annotated treatment and clinical outcome information.

We make extensive use of grid plots, which help visualize the clonal architecture of aneuploid tumor samples and provide visual feedback on the absence or presence of bias in segmented CN data. We also describe the key issues and challenges in CN estimation of subclonal samples, and show how local subclonal integer CN estimates are vital for correct classification of mutations.

Our demonstrations are restricted to a handful of the 52 RESPONSIFY *HER2*-positive breast cancer samples. Complete analyses of all samples with medical results, including potential biomarkers for resistance to trastuzumab-based therapy, will be published separately.

Our results are divided into three parts (A to C). In part A we present grid plots and demonstrate key issues in the estimation of CN of subclonal tumor samples in a simulated setting, to show that even with no noise or bias, subclonal chromosomal CNs can only be estimated in some genome segments, in samples with simple subclonal architectures, and even then relying on subjective assumptions. In part B, still in a simulated framework with no noise or bias, we show how the subclonal chromosomal CNs play a vital role in the classification of mutations as clonal or subclonal. In part C we briefly discuss our data. We suggest a probabilistic strategy to separate subclonality from noise in segmented CN data, and to assign a clonality status to a mutation. We also supply a two-dimensional grid rotation method to adjust for B allele fraction bias, which is common in our datasets.

We will refer to the number of chromosomal copies at a genome position in specified cells as their (true) integer CN. The average integer CN across cells from a tumor sample at a genome position will be called the (true) average CN. SNP array signals, which have been preprocessed, segmented and possibly normalized towards germline array data so that they are supposedly proportional to average CNs apart from noise deviations, will be called array CNs. By cell fraction we mean the percentage of sample cells (out of both normal and tumor cells of a sample) that make up a specified subclone.

## Results

### A: Grid patterns and integer CN estimation in simulated aneuploid tumors

In this section we present grid plots and demonstrate key issues in the estimation of CN of subclonal tumor samples in a simulated setting, to show that even with no noise or bias, subclonal chromosomal CNs can only be estimated in some genome segments, in samples with simple subclonal architectures, and even then relying on subjective assumptions. This step is important for subsequent classification of mutations as clonal or subclonal, since the mutation VAFs depend on local integer CNs in the tumor cells.

#### Clonal tumors and grid plots

**Figure 1:**
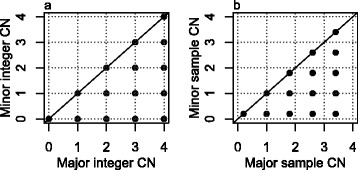
**Grid plot of a simulated clonal tumor sample. (a)** Grid plot of the integer CNs of a simulated single aneuploid tumor cell where all the combinations of major and minor integer CNs from 0 to 4 occur in the genome. **(b)** Grid plot of the average CNs from a simulated tumor sample with purity 80% and clonal CNs as in (a). Note how this grid is a version of (a) shrunken towards the point (1,1).

A normal, diploid cell has one copy of each parental chromosome in its nucleus. We say its integer CNs are (1,1). Aneuploid tumor cells exhibit integer CNs other than (1,1), including segments with loss of heterozygosity (LOH), such as (0,1) or (0,2), or CN gains, such as (1,2), (1,3) or (2,2). Each genome segment of constant CN in an aneuploid tumor cell may be represented by a point in a grid plot, a figure which displays all the combinations of CNs that occur throughout the genome in that cell, in a minor (smaller) versus major (larger) homologue CN pattern (extending the idea of TAPS plots in [21]). Figure 1a is a grid plot of simulated integer CNs in a cell in which each possible major and minor combination of 0, 1, 2, 3 and 4 copies occurs somewhere along the genome.

Tumor samples consist of thousands of tumor cells plus an unknown fraction of normal diploid cells, which we call normal contamination. We simulate a sample with clonal tumor CNs (identical integer CNs across all the tumor cells) as in Figure 1a with fraction (purity) *p* of tumor cells. Each average CN *e* of a given homologue and genome segment will then have the form:

1 $$ e=\left(1-p\right)+pc,\;c=0,\;1,\;2, \dots,\;4, $$

where *c* is the integer CN in the tumor cells (grid plot in Figure 1b). Compared with Figure 1a, each point in Figure 1b is shifted (shrunken) towards the point (1,1), since each average CN is the average of the integer CNs (1,1) of the normal cells and the tumor cell integer CNs.

Integer CNs and the purity of a tumor sample can only be unambiguously estimated from the unbiased, noise free average CNs via Equation 1 if 1) the sample is known to be clonal, and 2) there are at least two points in the grid plot for which the difference is known on an integer CN scale. For example, it may be known that two consecutive vertical grid points reflect a difference of one copy in the minor homologue.

With tumor samples, it is seldom known that a sample is clonal (1), so we broaden the CN estimation framework to that of (potentially) subclonal tumors.

#### Subclonal tumors

For tumors with heterogeneity, CN estimation comes down to estimation of the cell fraction and integer CNs of each subclone. As we shall see, this is a very difficult task with the data we consider.

**Figure 2:**
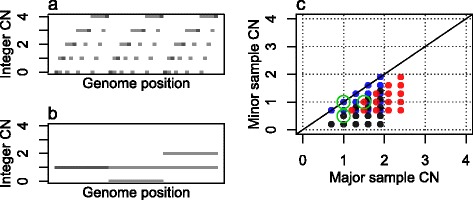
**Grid plot of a simulated, subclonal tumor sample. (a)** Genome integer CNs in the aneuploid subclone A. **(b)** Genome integer CNs in the less variable subclone B. **(c)** Grid plot from sample with 20% normal cell contamination, 30% cells from subclone A and 50% cells from subclone B. In grid plots each data point represents average CNs of a genome segment. Different colors represent genome segments with different behaviors in terms of their average CNs across the sample cells. In this grid plot each third of the genome results in a separate grid pattern (blue for subclone B integer CNs (1,1), black for (0,1) or red for (1,2)) their size determined by the fraction of subclone A cells. The three grids are positioned in a larger grid for which the size is determined by the larger fraction of subclone B cells (green).

Grid patterns from tumor samples with heterogeneity are more complicated than those in Figure 1. We simulate a sample consisting of 20% germline cells (*p* =80% purity), *α* =30% cells forming an aneuploid subclone A with integer CNs as in Figure 2a and *β* =50% cells forming another subclone B with integer CNs as in Figure 2b. Simulated average CNs are segment-specific averages of the subclonal integer CNs across all the sample cells, so in a given genome segment they take values of the form:

2 $$ e=\left(1-p\right)+\alpha {c}_a+\beta {c}_b, $$

where *α* + *β* = *p* and *c*_*a*_ and *c*_*b*_ are the integer CNs of the subclone A and B cells in that segment. The grid plot for the sample in Figure 2c consists of three small grids, each of which originates from the CNs in A combined with the CNs in one of the three B segments. The size, or rather the density, of the small grids is due to the small fraction of cells in A. The positions of the small grids follow that of a more sparse grid, determined by the larger fraction of cells in B. Alternatively, the grid plot could be seen as many three-point sparse grids (the green circles being one of them), positioned according to the denser pattern of subclone A.

For one subclone (say A), the cell fraction (*α*) can be estimated from the perfect, noise-free average CNs via Equation 2 if *Condition 1:* There are at least two points in the grid plot for which the difference is known (on an integer CN scale) and known to be due only to a change of integer CNs in subclone A (so that all other subclones have constant CNs throughout these two segments).

Given the cell fraction of subclone A, its integer CNs can be estimated from unbiased, noise-free average CNs via Equation 2 (or its extensions to more than two subclones) if *Condition 2:* The integer CNs and cell fractions of all subclones other than A of the sample are also known.

**Figure 3:**
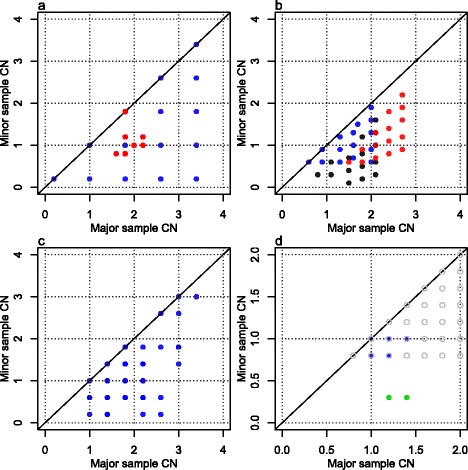
**Grid plots of simulated tumor samples with different subclonal architectures. (a)** Aneuploid tumor with further subclonality in a small part of the genome. **(b)** Tumor with three subclones, all with CN alterations. **(c)** Tumor with two subclones of equal size. **(d)** Tumor with two subclones of different sizes. Note that each data point in a grid plot represents the average CN across all sample cells. Different colors do not represent different subclones, but highlight specific parts of the genome which we discuss further in Materials and methods.

Condition 2 seems to be a catch 22 in that no subclonal integer CNs can be estimated without knowing the integer CNs of the other subclones, but there is an important exception. If the grid pattern suggested by condition 1 includes the point (1,1), the integer CNs of all other subclones are normal (1,1) in the genome segments of the points for which condition 1 is true. However, condition 1 is seldom truly known for any points (Figure 3a). Therefore, integer CN estimation in subclonal tumor samples can only be done from noise-free average CNs if the sample has certain properties and under certain assumptions. In Materials and methods we further demonstrate CN estimation challenges caused by selected subclonal structures through Figure 3, and outline properties and assumptions under which subclonal CNs can be estimated.

#### Purity versus cell fraction

CN alterations in tumor cells appear diluted in average CNs because of the germline (normal) cells in the sample (Figure 1), which are always present. If a sample is known to be clonal, the purity of the sample can be deduced from the density of an observed average CN grid pattern via Equation 1: the distance between consecutive grid points is equal to the purity. However, when we study tumor samples we usually do not know whether or not that sample is clonal. In this case, as acknowledged by Durinck *et al*. [2], further, indistinguishable dilution occurs when CN alterations are present only in part, but not all, of the tumor cells (Figure 2). With or without heterogeneity, the density of a grid pattern in a grid plot holds information about the cell fraction which express CN alteration throughout some genome segment(s) in which other subclones have constant CNs: the distance between consecutive grid points is equal to that cell fraction (Equation 2). Average CNs do not carry sufficient information to deduce sample purity, although it is sometimes suggested that they do [1,22].

#### Scaling: where is (1,1)?

**Figure 4:**
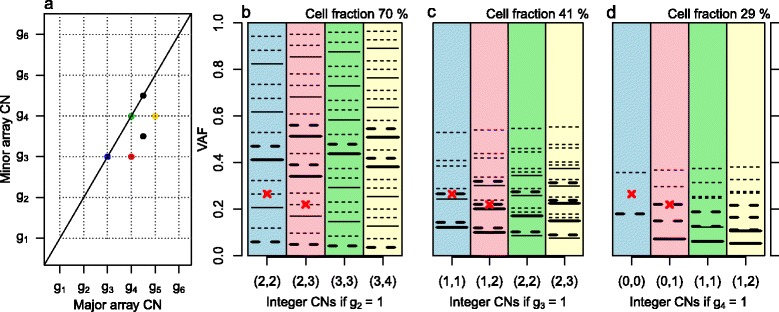
**Grid plot and expected VAF levels of simulated tumor sample. (a)** Grid plot of simulated, noise- and bias-free array CNs. The scale of the array CNs is unknown. The four colored points suggest the grid pattern drawn for subclone A, but it is unknown whether (1,1) integer copies happen at (*g*_2_, *g*_2_), (*g*_3_, *g*_3_) or (*g*_4_, *g*_4_). **(b-d)** The three scenarios are illustrated., Each scenario has one colored column for each of the colored genome segments. Two of the segments have mutations on them (red crosses), with VAFs as shown on the y-axes (simulated without noise or bias). The different scalings suggest different integer CNs (*c*_1_, *c*_2_) of the segments (labels on x-axes), which give different potential expected VAF levels under certain assumptions (horizontal lines). The mutation on the blue segment does not fit any suggested VAF level in (d), suggesting *g*_4_ ≠ 1. Assuming that some VAF levels are more plausible than others (thick rather than thin horizontal lines) also rules out (b) in favor of (c): *g*_3_ = 1 (see Materials and methods).

Summarizing the preceding discussion, cell fractions and integer CNs can be quantified from unbiased, noise-free average CNs for some subclones and for some genome segments, if the tumor sample has some fortunate properties and we rely on a set of assumptions. Array CNs are at best proportional to the average CNs in the sample hybridized. Even if they were noise and bias free, array CNs are insufficient for determination of an identified subclone’s integer CNs, its cell fraction and the scaling factor without further information [1,22,23]. The colored points in the simulated grid plot of Figure 4 appear in a regular grid pattern as marked by dotted lines, but it is unknown which lattice point corresponds to integer copies (1,1): (*g*_2_, *g*_2_), (*g*_3_, *g*_3_) or (*g*_4_, *g*_4_)? Each of the colored points must have at least 0 minor integer copies. Therefore, the grid pattern suggests that g_4_ is at least 1, or that (1,1) falls no higher than (*g*_4_, *g*_4_). In Materials and methods we explain how the three proposed (1,1) scenarios originate from different integer CNs, cell fractions and scaling factors but result in identical array CNs, or, equivalently, identical total (minor + major) array CNs and B allele fractions.

Pounds *et al*. [23] suggest solving this issue by identification of genomic regions with normal CNs (RAP, reference alignment procedure), which may be possible for some samples but not for all. In the context of heterogeneity, VAFs can sometimes provide sufficient information, and these are used in the software Absolute [1] together with database knowledge about chromosome arm level CN alterations in common cancer types. Carter *et al*. [1] and Pounds *et al*. [23] both stress that manual care with each sample is vital for correct CN estimation. We examine circumstances under which knowledge of overall sample ploidy, matched normal sample array CNs or VAFs can resolve the scaling issue below.

##### *Ploidy can sometimes help*

**Figure 5:**
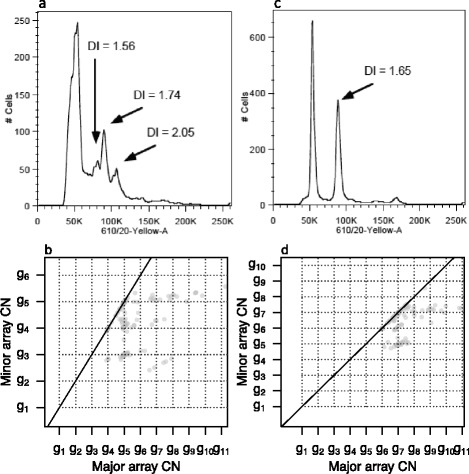
**FACS ploidy profiles.** FACS ploidy profiles (upper panels, number of cells versus cell ploidy) and grid plots (lower panels) of three actual data samples. Peaks at 50 K in the FACS profile correspond to diploid cells. **(a)** Sample 11 has multiple subclones suggested by multiple peaks in the FACS profile. Some peaks comprise approximately the same cell fraction (peak height). **(b)** A grid pattern appears in the sample 11 grid plot, but since the FACS profile reveals several subclones of the same size, assumption 1 is not reasonable and integer CNs cannot be located to specific subclones: CN alteration which agrees with the lattice points could originate from any of the subclones of the corresponding size. **(c)** The sample 29 FACS profile suggests that the largest non-diploid subclone has ploidy 3 to 4. **(d)** The sample 29 grid plot suggests a subclone with ploidy 3.05 if *g*_6_ = 1, ploidy 5.05 if *g*_5_ = 1, ploidy 7.05 if *g*_4_ = 1, and so on. The FACS profile and grid plot thereby together suggest that *g*_6_ = 1 for sample 29. DNA Index (DI) is a measurement of ploidy.

A sample’s overall ploidy is the sample’s average (minor + major) integer CN across the genome and across all subclones. In Materials and methods we explain how an independent overall ploidy estimate (for example, from a fluorescence-activated cell sorting (FACS) run) may help us resolve the true position of (1,1). Often, overall ploidy estimates are not given by FACS, but with samples having simple subclonal architecture we may compare subclone-specific ploidies estimated for each potential position of (1,1) (Materials and methods) to the suggested subclone ploidies from FACS, and deduce the true position of (1,1). Figure 5 shows FACS ploidy profiles and segmented SNP array data grid plots for two samples. Sample 11 (Figure 5a,b) has several subclones and integer CNs cannot be located to specific subclones. Sample 29 (Figure 5c,d) has most of its CN alteration in one subclone and the FACS and grid plots combined give clues to the scaling of array CNs.

##### *Paired normal SNP array normalization helps in theory*

**Figure 6:**
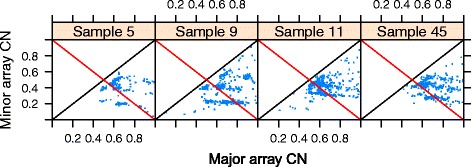
**Array CNs normalized towards matched normal tissue SNP array CNs for four actual data samples.** Theoretically, points in a cluster around the intersection of allelic balance (black line) and total array CN equal to 1 (red line) should reflect segments with integer CNs (1,1). In practice, the cluster closest to the intersection may reflect (1,1) (samples 5 and 9) but not always (samples 11 and 45).

If array CNs have been normalized towards matched normal tissue SNP array CNs, segments with minor + major array CNs equal to 1 (red lines in the example samples of Figure 6) and allelic balance (black lines in Figure 6) - that is, segments at the intersection of the two lines - should theoretically correspond to normal integer CNs (1,1). Several CN packages (SOMATICS [24], PICNIC [25], SiDCoN [26], GAP [27] and ASCAT [22]) rely on normalized array CNs and assume the solution with the minimal possible CNs that fit their (grid) pattern. ASCAT notes that they go wrong if that assumption is not correct. A look ahead at our actual data grid plots (Figure 6) suggests that this method will not work in general for our samples.

##### *VAFs can sometimes help*

For samples with a reasonable amount of mutations in ‘informative’ locations, VAFs can help deduce the scaling of array CNs if we rely on a set of chosen assumptions. We outline such a framework in Materials and methods through Figure 4.

#### Estimation of cell fraction, integer CNs and average CNs

The cell fraction of subclone A and its integer CNs in genome segments that coincide with the subclone’s lattice points (type A segments) can be estimated under the fortunate circumstances described, given the properly scaled array CNs. Let (*g*_*normal*_, *g*_*normal*_) be the position of (1,1) and Δ the distance between two consecutive grid lines. Then we can derive the cell fraction *α* = Δ/*g*_*normal*_ of subclone A and its integer CNs *c*_1_ = (*a*_1_ − *g*_*normal*_ − Δ)/Δ and *c*_2_ = (*a*_2_ − *g*_*normal*_ − Δ)/Δ, where ${\overset{\rightharpoonup }{a}}_j=\left({a}_1,{a}_2\right)$ are minor and major array CNs of unknown scale. The average CNs can be derived as *e*_1_ = *a*_1_/*g*_*normal*_ and *e*_2_ = *a*_*e*_/*g*_*normal*_.

### B: Clonal or subclonal mutations

With cell fractions and integer CNs of one or more subclones resolved and with knowledge of the sample purity, we can assess whether a VAF suggests the corresponding mutation is clonal (present in all tumor cells) or subclonal (not present in all tumor cells) if we rely on previously outlined and further properties and assumptions (see Materials and methods).

The simulated sample of Figure 4 provides an example. Figure 4c gives the integer CNs and cell fraction of the sample’s main subclone A. The genome segment of the blue grid point (Figure 4a) has no CN alteration in any cells. The blue column in Figure 4c shows expected VAF levels of heterozygous mutations present only in the cells of the main subclone (thick continuous horizontal line), present only in all other tumor cells (bottom thick dashed line) or present in all cells (top thick dashed horizontal line) of such genome segments. The observed VAF (red cross) coincides with the latter, so the corresponding mutation is estimated to be clonal. The red genome segment (Figure 4a) has CNs (1,2) in the main subclone, and normal CNs in all other cells. The pink column of Figure 4c shows the expected VAF levels given these CNs (thick horizontal lines). The top two thick dashed horizontal lines reflect expected VAF levels of clonal heterozygous mutations present on all its homologue’s copies. The mutation on this segment is hence estimated to be clonal too. If it had coincided with one of the lower horizontal lines, we would have estimated it to be subclonal.

The four colored columns of Figure 4c show different expected VAF levels of clonal mutations (top one or two thick, dashed horizontal lines, one or two depending on whether the minor and major integer CNs are equal or not), and different expected VAF levels of mutations present only in the main subclone A (thick, solid horizontal lines), resulting from different local integer CNs. We also note that other cell fractions of subclone A, together with other integer CNs (Figure 4b,d), would give other expected VAF levels. Two important conclusions follow. First, in order to enable classification of mutations as clonal or subclonal from VAFs with any precision, correct estimation of subclonal integer CNs and cell fractions is vital. (The procedure will still rely on simplifying assumptions, even for mutations on fortunate segments on grid plot lattice points of an identifiable subclone, and when there is no noise or bias in VAFs or segmented CN data.) Second, one subclone is associated with a whole set of expected VAF levels, dependent on the subclone’s cell fraction and integer CNs, for example, the thick continuous horizontal lines in Figure 4b-d. This contrasts with what has sometimes been suggested [8]. We return to this point in the Discussion.

### C: Data examples

In this section we illustrate what we learned in the previous sections through selected analyses of single tumor and matched normal samples from a set of 52 newly diagnosed *HER2*-positive breast cancer tumors. The patients were all part of a European Union funded project (RESPONSIFY) investigating biomarkers of resistance to trastuzumab plus chemotherapy, which is standard treatment for newly diagnosed breast cancers that have *HER2* amplification. Clinical follow-up data are available for each patient through a median time of 5 years, including relapse status. SNP arrays and WES were run as described in Materials and methods. Tumor sample purities (fractions of tumor cells) were estimated by a pathologist. The median purity was 87% across tumors. For details about SNP array preprocessing and detection of mutations, see Materials and methods.

#### Bias in BAF can cause skewness in SNP array data

**Figure 7:**
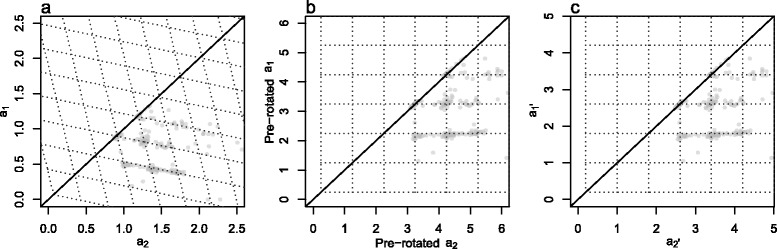
**Sample 45 grid plots.** For the description of notation see Materials and methods. **(a)** Original array CNs with skewness. **(b)** Array CNs after pre-rotation in search of start values (see Materials and methods). **(c)** Array CNs after completed grid rotation, which removes the skewness. Unless otherwise stated, we refer to array CNs as rotated array CNs and we drop the prime from ${\overset{\rightharpoonup }{\mathrm{a}}}^{\hbox{'}}$.

Our observed array CN grid plots display skewness (Figure 7), so that segments with the same minor CN appear in clusters on a sloping rather than a horizontal line, and segments which have the same major CN appear in clusters on a sloping rather than a vertical line. This phenomenon is particularly pronounced in those of our samples that do not have matched normal sample SNP array data. This is an artifact caused by a systematic bias in our SNP array BAFs which needs to be removed in order to make the CN estimates comparable to WES VAFs. We do this by grid rotation and describe the BAF bias (see Materials and methods). Unless otherwise stated, we refer to array CNs as rotated array CNs.

#### *A typical* HER2+ *grid plot*

**Figure 8:**
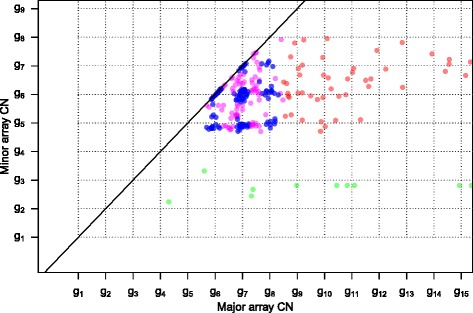
**Sample 5 grid plot.** The small, regular grid suggests that a low fraction of the sample cells form an aneuploid subclone (A). The segments with minor array CNs far below the regular grid may be caused by LOH in a different, larger subclone. Segments are classified into types A (blue, regular grid pattern), B (pink, breaking regular grid pattern), C (green, lower array CNs than the most evident grid pattern) and D (red, high enough array CNs for regular grid patterns not to appear).

Most of our *HER2*-positive breast cancer sample grid plots are similar to Figure 8, which suggests that they have an aneuploid subclone A in a small fraction of cells (because the regular grid pattern is small). Many of the grid plots also have some short segments with minor array CNs below the most prominent grid pattern, as in Figure 8, which suggests that a larger subclone than the aneuploid one has some LOH.

#### Probabilistic model to separate subclonality from noise and a simple endpoint quantifying ITH

In Figure 8 we identify a regular grid pattern (type A segments, blue), possibly caused by CN variation in a subclone, say A, of cells. We also spot array CNs that do not follow the grid pattern, in between the regular lattice points (type B segments, pink). In general we see lattice points (type A) as the default location of grid plot points, and it is only if we observe significant evidence to the contrary that we set the type of a segment to B according to the following process.

**Figure 9:**
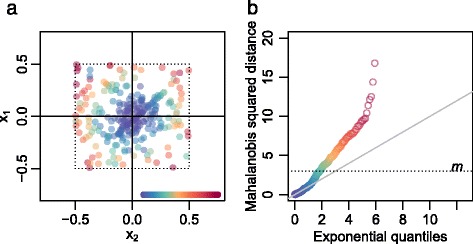
**Noise or heterogeneity in sample 5. (a)** Positions of array CNs relative to their closest grid plot lattice points, with coloring by statistical distance. Note that positions have been scaled to fit the lattice point and its closest segments within ±0.5. **(b)** Exponential(½) *qq*-plot with the same coloring as (a). Points above the horizontal cutoff (*m*) indicate segments with array CNs significantly different from the lattice points (type B segments).

The classification between type A and B segments is made through the two-dimensional distribution of grid points $\left\{{\overset{\rightharpoonup }{a}}_i\right\}$ relative to their closest lattice points $\left\{{\overset{\rightharpoonup }{e}}_i\right\}$, in effect overlaying all the lattice points into $\left\{{\overset{\rightharpoonup }{x}}_i={\overset{\rightharpoonup }{a}}_i-{\overset{\rightharpoonup }{e}}_i\right\}$ (Figure 9a). We fit a two-dimensional *t*-distribution [28] centered at the origin to the $\left\{{\overset{\rightharpoonup }{x}}_i\right\}$, with maximum robustness (degrees of freedom =2) in order to capture the variation of observations in the dense central cluster (which may truly have CN alteration in subclone A only) but not that of the many outliers (which may not originate from CN alteration in subclone A). The estimated covariance matrix *Q* is used to calculate a segment length-weighted squared Mahalanobis distance ${M}_i^2={\overset{\rightharpoonup }{x}}_i^T{\left(Q/{w}_i\right)}^{-1}{\overset{\rightharpoonup }{x}}_i$ for each segment *i*, which should follow an exponential distribution with scale parameter ½ for segments within the dense centre cluster. We choose a cutoff *m* where the linearity in the exponential *qq*-plot starts to fail (Figure 9b; commonly conservatively chosen to *m* =3), and classify segments as type A if ${M}_i^2\le m$ and type B otherwise. The segment length weight ${w}_i=1-{e}^{-{l}_i/500000}$, where *l*_*i*_ is the length of segment *i*, downweights ${M}_i^2$ values of short (<1 Mb) segments, since we think their deviance from the origin may be due to noise in the array CNs of such small segments, rather than to a true pattern-breaking deviance in CNs.

The fraction of the genome covered by type B segments out of that covered by type A and B segments is a simple measure of the amount of ITH in a sample. This endpoint estimates the fraction of the genome in which the sample has CN alteration in other subclones than the main subclone A (possibly in addition to CN alteration in A). It has proved useful for prediction of relapse in the RESPONSIFY samples. Details will be published separately.

#### Scaling: resolving location of (1,1) with help of VAFs and purity

We estimate the scaling of a sample’s array CNs by the scenario that best fits VAFs estimated from WES data for mutations in segments classified to be of type A with respect to the sample’s most evident subclone A. By our assumptions, these segments have CN variation only in the subclone A cells. Out of the 52 RESPONSIFY samples the scaling was resolved in this manner for 48 samples.

**Figure 10:**
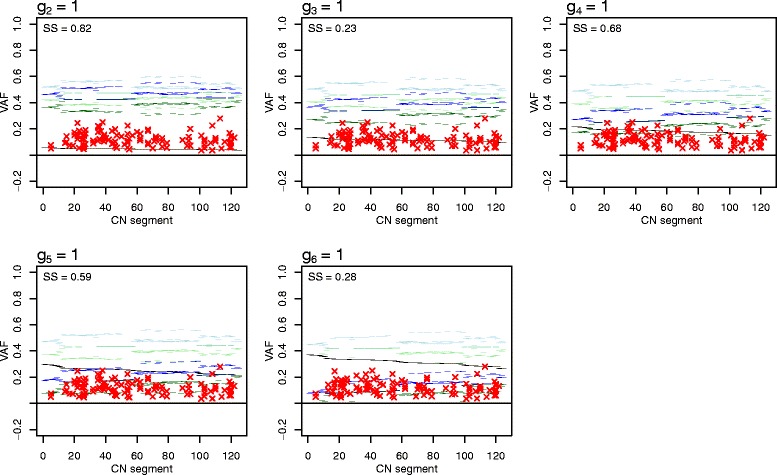
**Sample 5 VAFs compared to expected VAF levels.** As in Figure 4b-d, each panel shows observed VAFs (red crosses) and expected VAF levels given a potential position of (1,1) in the sample grid plot. Expected VAF levels are all under assumption 6, with mutations on the minor allele of subclone A expected on green VAF levels, on the major allele of subclone A expected on blue, on the single copy of all tumor cells not in A expected on black (the purity of this sample was estimated to 84%), clonal mutations on minor allele on light green and clonal mutations on major allele on light blue horizontal lines (see assumptions 3 to 5 in Materials and methods). Genome segments have been ordered by decreasing expected VAF if present only on non-A cells and by increasing (minor + major) array CN (x-axis). SS gives the sum of squared distances from each observed VAF to its closest expected VAF.

**Figure 11:**
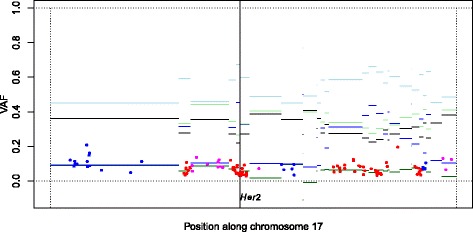
**Sample 16 VAFs fit expected VAFs along chromosome 17.** Sample 16 VAFs (points) and expected VAF levels (horizontal lines; Figure 10) along chromosome 17 given subclone A CN estimates and its sample cell fraction *α*. This sample has 1,232 detected mutations, which is many more than the median 156 of the 52 RESPONSIFY samples. We see an almost perfect fit of the observed VAFs to those expected. For color coding of VAFs, see text. This figure also shows how the mutation rate differs across the chromosome, a different type of heterogeneity studied by Lawrence *et al*. [29]. The purity of this sample is unknown and is imputed to 90%.

In Figure 10 we display a typical example rather than a perfect one (as, for example, that of Figure 11 below). Each panel shows the five expected VAF levels (y-axis, horizontal lines with different colors) for each type A CN segment (x-axis, ordered by decreasing expected VAF if present only on non-A cells and by increasing minor + major array CN) for one potential position of (1,1) of the sample introduced in Figure 8 under assumptions 3 to 6 in Materials and methods. The observed mutation VAFs of type A segments are shown as red crosses. Each panel also gives SS, the sum of squared distances to each VAF’s nearest expected VAF level. The figure suggests that *g*_3_ = 1 or *g*_6_ = 1, since in these panels the observed VAFs are, on average, closer to their expected levels (they have lower SS than the other panels). Note that we do not expect all mutations in type A segments to follow our assumptions and fit one of the expected levels, but we assume that most mutations do, in order to resolve the scaling of the array CNs.

**Figure 12:**
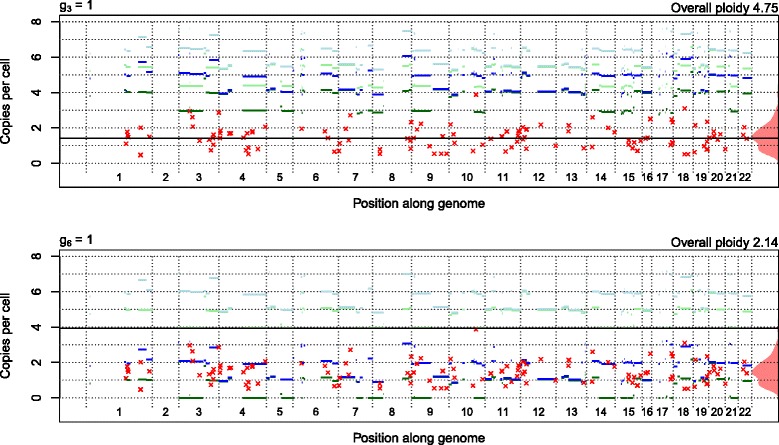
**Mutation multiplicities compared to subclonal CNs.** Sample 5 VAF fits to array CN data of type A segments and their mutations for the two most plausible positions of (1,1): *g*_3_ = 1 or *g*_6_ = 1. Each scenario results in different minor (green) and major (blue) subclone A integer CN estimates, and multiplicities of the mutations if these sit on A cells only (red). Light and black horizontal lines show expected y-levels of clonal mutations and mutations present on only non-A cells, all according to assumptions 3 to 6. For details, see text.

To further differentiate between the two suggested scenarios we transform these panels’ y-levels to show subclone A integer CN estimates under these scenarios (Figure 12), also showing the segments by their genome position. Now, the (dark) green and blue horizontal lines are the minor and major integer CN estimates of subclone A for segments that have CN alteration only in subclone A. The red crosses’ y-levels show the mutation multiplicities (calculated under the assumption that they sit on A: $\frac{D}{\alpha }VAF$ ), which equal an integer CN estimate (green or blue horizontal line) if the mutation VAF equals the corresponding expected VAF level. The black line shows the expected y-level of the multiplicity for mutations on the single copy of all tumor cells not in A, which is equal across all segments. The light green and light blue horizontal lines show the expected y-levels of clonal mutation multiplicities. We see that if *g*_3_ = 1, subclone A has no single allele integer CN below 3, a rough calculation (see Materials and methods) suggests the sample has overall ploidy above 4, and all mutations seem to sit only on the assumed diploid cells (black horizontal line), not in subclone A. If *g*_6_ = 1, subclone A has single allele integer CNs from 0 and above, the rough overall ploidy estimate is just over 2 and most mutations seem to sit on the subclone A cells. The majority of our samples end with a similar choice. The *g*_6_ = 1 scenario sounds more reasonable and therefore we choose to proceed with that. When in doubt we choose the conservative scenario with the smallest integer CNs and smallest size *α* of subclone A.

See Materials and methods for our suggested estimation of subclonal architecture, cell fractions and integer CNs.

#### Clonal or subclonal mutations

Our classification of mutations as clonal or subclonal is based on the methods outlined for simulated data. To acknowledge the uncertainty of real VAFs we run a set of non-inferiority, inferiority and equality tests for each VAF based on its binomial two-sided 90% confidence interval (CI) from the sequencing number of variant versus reference reads. For details, see Materials and methods.

#### Clonal or subclonal CN alterations

The vertical continuous line in Figure 11 denotes the position of the *HER2* (*ERBB2*) gene. Our samples have been diagnosed as *HER2*-enriched, and they do have a high, type D, major array CN at this position. Unfortunately no samples have VAFs that match the major homologue, so it is not possible to assign the clonality status or subclonal origin of *HER2* enrichment. The number of detected mutations in the 52 RESPONSIFY samples varies from 1 to 1,232 (median 156.5), and only a handful of samples have enough mutations (say >900) to enable assessment of subclonal origin of CN alteration on a large scale.

## Discussion

The aim of this paper is to highlight challenges in CN estimation that influence mutation classification but are infrequently acknowledged in the literature as well as propose solutions that may aid in the quantification of ITH in tumor samples that have high levels of CN alteration. We have demonstrated how even with no noise or bias, integer CNs of tumor samples with ITH can only be estimated from segmented CN data in samples with a simple clonal architecture, given further information from, for example, WES VAFs or FACS images, and under a series of assumptions. Even with such samples, integer CNs can only be deduced for some subclones and only across a subset of the genome.

Classification of mutations as clonal or subclonal further requires knowledge of the sample purity, which cannot be deduced from segmented CN data. The classification relies on comparing observed VAFs to expected VAF levels given purity, subclonal cell fractions and local CNs. Therefore, the assumptions made in the CN estimation procedure will have a large influence on how mutations are classified, and on how the results can be interpreted.

We have also suggested a simple ITH endpoint for tumor samples with a high level of CN alterations based on segmented CN data alone and which does not require knowledge of subclonal cell fractions or integer CNs.

### Estimate average CNs from sequencing

We have used SNP arrays to derive segmented CN data for the RESPONSIFY samples. Alternatively, sequencing depths could be used [12–17], which has the advantage that it works well on formalin-fixed paraffin-embedded tissue, whereas SNP arrays usually require frozen tissue, which is less practical to validate. The expected (true) average CN patterns of tumor samples with heterogeneity are the same whether average CNs are estimated from SNP arrays or sequencing data. The challenges presented hence apply either way: there is ambiguity between purity and heterogeneity, there are difficulties deducing subclonal structures and assigning a subclonal origin to a segment with CN alteration, and the scaling of array CNs or SNP position sequencing depths relative to average CNs is unknown. Both sequencing and SNP array data may suffer from bias which needs attention before estimation of average CNs, although the types of bias are different. There may be BAF bias in SNP array data and GC bias in sequencing depths. Standardizing tumor sample sequencing depths to matched normal sample sequencing depths comes with challenges that are different from those of standardizing tumor sample array CNs to matched normal sample array CNs. We generally seek more evidence that the results after different steps of analysis look plausible than is typically presented in a literature dominated by model-based inferences. We find that just as important as detailed model descriptions. As for CN determination, a study of the two-dimensional grid plots (applicable equally well to SNP array CNs and sequencing depths) of average CN estimates can help reveal bias and give clues to sample architecture.

### Whole genome sequencing versus whole exome sequencing

WGS identifies many more mutations than WES (which can only find mutations in gene exons), but is comparatively more expensive. More mutations help in assigning CN alterations to identified subclones, and resolving the scaling of segmented CN data in relation to average CNs. Therefore, WGS is generally a benefit for assessment of integer CNs, clonality of mutations or phylogenetic trees (see below) in subclonal tumor samples.

### Phylogenetic trees

Given a set of identified subclones in a sample, trees can be inferred by assigning mutations to subclones and checking whether mutations close in genomic location but assigned to different subclones tend to co-appear or never co-appear on the same fragment. Co-appearance indicates that one of the subclones is in turn a subclone of the other, and no co-appearance indicates that the subclones belong to independent branches of the tree. Given that WGS identifies more mutations than WES, WGS is again a benefit. Since the majority of our samples have too complicated subclonal structures for more than one or two subclones to be identified in detail, and relatively few mutations identified by WES, detailed phylogenetic trees are not generally within reach. The number of identified mutations in the 52 RESPONSIFY samples ranges from 1 to 1,232 (median 156.5; samples were selected so that they had at least one identified mutation). The WES average coverage of the samples ranges from 25 to 179, with median 108.

### Clustering of cancer cell fractions

It has been suggested [8,9,12–17] that, with WGS, subclones can be identified via groups of mutations present in similar fractions of cancer cells. On this topic we would first like to stress that clustering of a sample’s VAFs is something different from clustering of the sample’s cancer cell fractions. The former may cluster because of aneuploidy in the sample, even if the sample has no heterogeneity: a sample with aneuploidy has several expected VAF levels (like the thick continuous horizontal lines in Figure 4c), so each subclone corresponds to several VAF clusters. Also, different subclones may have overlapping expected VAF levels.

To the best of our knowledge, Papaemmanuil *et al*. [9] do not take local CNs into account when classifying mutations as clonal or subclonal. They assume that the mutations with the highest VAFs are clonal, and classify mutations as subclonal if their CIs do not overlap with those of the ‘clonal’ mutations. As seen in Figure 11, expected clonal VAF levels (light green and light blue horizontal lines) may be very close to expected subclonal VAF levels (black horizontal lines). Therefore, we do not generally recommend classification of mutations by comparing a sample’s VAFs within themselves with no reference to local integer CNs.

Nik-Zainal *et al*. [8] estimate the cancer cell fraction (ccf) of each mutation by what we call the ‘multiplicity’ of the mutation given integer CN estimates in the most evident identified subclone. For RESPONSIFY sample 5, this is exactly the y-levels of mutations in Figure 12. More precisely, Nik-Zainal *et al*. [8] estimate ccfs as the minimum of the multiplicity and 1, and look for clusters among the mutations with ccf <1. We see no clusters among the y-levels of mutations below the dotted horizontal line of 1 in Figure 12, but perhaps we have too few mutations of type A detected from the WES data. We note that such clusters would only reveal very small subclones with low integer CNs and daughter subclones of the most evident identified subclone. We also note that with this ccf estimator, mutations of such a small subclone will get different ccf estimates if they sit on segments with different integer CNs in the most evident identified subclone, so several clusters may arise from the same subclone. Nevertheless, this method may help screening for long subclonal CN alterations to be verified by phasing of SNPs and mutations on the same sequencing reads, which is what Nik-Zainal *et al*. [8] do.

The PyClone algorithm [14] clusters mutations on the basis of their VAFs corrected for local CN, termed the ‘cellular prevalence.’ To do so, at each mutation the algorithm splits the cells in the sample into the ‘normal population’, the ‘reference population’, consisting of all cancer cells which do not contain the mutation, and the ‘variant population’, consisting of all cancer cells with the mutation. It makes a ‘key assumption’ that all cells within their three populations have the same genotype. We have applied the algorithm to the six samples discussed and made available in this paper. It produces an estimate of the number of subclones in a sample, and assigns mutations to subclones. The results are, in part, consistent with, but also complementary to, ours, bearing in mind that we do not attempt to estimate the number of subclones in a sample. For example, sample 16 depicted in Figure 11 has 1,232 somatic mutations, and PyClone infers 6 clusters, assigning over 900 to one and over 250 to a second. In data not shown, we inferred that CN alterations in a main aneuploid subclone only (segments of type A) comprised 90% of the genome and held 839 of the mutations (no others could be assigned to a specific subclone), while we found 8% of the genome to be segments of type B. This is a fair degree of consistency between rather different approaches to the same problem. On the other hand, sample 5 had its 199 mutations put into just 3 clusters by PyClone, but as can be seen from Figures 8, 10 and 12 it has a considerable amount of subclonality, and we see evidence of more than 3 subclones. Most of our samples are like sample 5 in being highly heterogeneous, and it seems likely that the differences between PyClone’s results and ours stem from a failure of their ‘key assumption’, in that we have different CNs between different subclones. This point is highlighted in [16], where it is noted that clonal inference using CN aberrations and B-allele frequencies need not be the same as that using somatic aberrations. Our approach and that of PyClone are different ways of integrating these two data types, while the integrative analysis of [16] is perhaps better than both if one has WGS data. Their method is not available to us as we do not have such data.

### CN estimation and mutation classification in the literature

Durinck *et al*. [2] identify CN neutral LOH regions within one tumor subclone and classify mutations as homozygous or heterozygous within the subclone. This aim is slightly different to ours, but the paper deserves a mention because it acknowledges that an identified CN pattern reflects CN alteration in a subclone rather than in all tumor cells.

The software Absolute [1] deduces integer CNs in pooled minor and major array CN histograms. The BAF bias of the RESPONSIFY array CNs cannot be spotted with one-dimensional histograms rather than two-dimensional grid plots, and in Materials and methods we demonstrate how Absolute therefore does not work with our samples. But given data without bias, Absolute estimates integer CNs under the assumptions that (i) only one pattern of equally interspaced peaks can occur, and (ii) the pattern reflects the clonal CNs of all tumor cells in the sample. With the theoretically expected CN patterns of Figures 2 and 3 as background we suggest this approach may be useful for samples with most CN alteration taking place in most of the tumor cells, and only small subclones (accounting for up to say 10% of the sample cells) expressing further CN alteration.

To deduce the scaling of the array CNs, Absolute suggests the scenario for which the majority of (all the sample’s) VAFs fit presence on one copy of one homologue of the large subclone. We acknowledge that this is different from our suggested scenario with VAFs (from the genome segments with CN alteration in the pronounced subclone) fitting presence on all copies of one homologue.

Nik-Zainal *et al*. [8] estimates integer CNs and sample purity with ASCAT [22], and thereby assumes the minimal CNs fitting array CNs (ignoring the unknown scaling) as well as assumptions (i) and (ii) above, as Absolute does. Again we suggest this approach may be useful for samples with most CN alteration taking place in most of the tumor cells, and only small subclones (accounting for up to say 10% of the sample cells) with other CN alteration. Nik-Zainal *et al*. [8] further refine the precise integer CN estimates with help of WGS depths at SNP positions. This may or may not eliminate any BAF bias in average CN estimates; a reader of the paper cannot deduce which. ASCAT fails with most of the RESPONSIFY samples, which are highly aneuploid and subclonal.

The methods also differ in their interpretation of mutations as clonal or subclonal. In simple terms we call a mutation clonal if its VAF (is larger than or) fits presence on all copies of one homologue in subclone A plus on one copy of one homologue in the rest of the tumor cells (a fraction of cells determined via the pathologist purity estimate). Mutations with significantly smaller VAFs we call subclonal. Absolute calls a mutation clonal if the VAF gives a high likelihood of its presence on at least one copy of a homologue of the (large) subclone. Mutations with a high likelihood of presence in less than one copy are called subclonal. Nik-Zainal *et al*. [8] similarly call a mutation clonal if it seems present in at least one copy of one homologue of the (large) subclone, except in segments with further subclonality (type B segments) where they require more. The methods will clearly classify mutations differently. Our method of calling clonal mutations is conservative, and will only find a few such mutations per sample (sometimes none, in particular since ambiguous mutations are not classified). The other three methods [1,8,9] are conservative with calling subclonal mutations and will only call those that are present in a small fraction of cells. To our knowledge there is no clear answer to which of these interpretations is more appropriate biologically.

## Conclusions

We have demonstrated that even with no noise or bias, integer CNs of tumor samples with ITH can only be estimated from SNP array data in samples with a simple clonal architecture, given further information from, for example, WES VAFs or FACS ploidy profiles, and only under a series of assumptions. Even with such samples, integer CNs can only be deduced for some subclones and only across a subset of the genome.

Estimation of local subclonal CNs has implications for the classification of mutations as clonal or subclonal. The classification also requires knowledge of the sample purity, which cannot be deduced from segmented CN data. The literature on this topic is divergent in assumptions and data analysis methods, with interpretational differences as a result. The insights demonstrated in this study impact research in heterogeneity and tumor evolution, with our emphasis being not only on data analysis methodology but also on the goals, design and interpretation of such studies.

We would like to stress the importance of illustrative figures to reveal bias and verify model assumptions in ITH studies. We think such evidence of performance is just as important as descriptions of analysis models in papers. As for CN determination, a study of two-dimensional grid plots of average CN estimates can help reveal biases and give clues to sample architecture.

## Materials and methods

This section provide further details and demonstrations of the points made in the main text.

### CN estimation challenges caused by selected subclonal structures

We aim to outline a set of assumptions under which subclonal cell fractions and integer CNs can be estimated from average CNs for some tumor samples. Let us first demonstrate some selected subclonal architectures with help from Figure 3.

The clonal, aneuploid tumor of Figure 1 would have average CNs as shown in blue in Figure 3a. We simulate a small subclone emerging from the tumor, so that part of a segment which originally had integer CNs (1,2) now splits up into small segments with different amounts of CN alteration relative to the original, main subclone. Figure 3a shows the resulting grid plot, in which the affected small segments have been colored red. We note that small subclones with additional CN variation to that of a main subclone will cause average CNs between (and sometimes even on top of) the main subclone lattice points.

Next, imagine a subclonal tumor with 90% purity, which has two subclones as in Figure 2 plus *γ* =10% cells forming another subclone C with integer CNs from 0 to 4, varying independently of the other subclonal integer CNs. Figure 3b shows simulated average CNs of such a tumor sample, where segments from each third of the genome has been colored differently. Presented with such a grid plot, the underlying subclonal architecture is not easily detected. Even if we were told the number of subclones (three), each average CN is a combination of three subclonal integer CNs, so integer CNs for individual subclones could not be estimated from average CNs alone. We note that the pattern of average CNs quickly gets out of hand as subclonality increases, and that average CNs between regular lattice points may not be caused only by small subclones that deviate from a main subclone (Figure 3a), but also by small subclones with integer CNs independent of those in a main subclone.

Even with only two subclones many samples cannot be resolved from average CNs. Figure 3c is a grid plot from a simulated tumor sample with two subclones of the same size, which have independently sampled integer CNs from 0 to 4. We note that even though one regular grid pattern can be identified in the grid plot, it is not necessarily caused by just one subclone.

A further difficulty is that in reality not all integer CN combinations will occur, and in particular not in combination with each integer CN in other subclones. Figure 3d shows the grid plot of a simulated sample with two subclones. Two separate regions of the plot show equally spaced grid points (blue and green). The blue points reflect segments with different integer CNs in the smaller of the two subclones, and (1,1) copies in the larger one. The possible lattice points on which such grid points can fall have been circled. The green points reflect segments with different integer CNs in the smaller subclone and (0,1) in the larger one.

### Properties and assumptions under which subclonal CNs can be estimated

In this section we describe some sample properties and assumptions under which conditions 1 and 2 hold so that cell fractions and integer CNs of subclones can be estimated. Imagine a tumor sample for which the following holds.

*Property 1*: The grid plot has a regular vertical/horizontal grid made up by at least two points. This indicates that there is CN alteration in a subclone or in all tumor cells throughout some genome segments where no other subclones have CN alteration. It may also result from the combined effect of CN alteration in two or more subclones. In order to proceed, we must simply assume (Assumption 1 below) that is not the case.

For example, the blue grid points of Figure 3d satisfy property 1. Under the following assumption, condition 1 holds so we can correctly identify a subclone (say A) in the tumor sample by its cell fraction.

*Assumption 1:* The regular spacing between the grid points of property 1 is caused by consecutive integer CNs in subclone A.

We now consider

*Property 2:* The point (1,1) is part of the grid pattern suggested by property 1, even if there are no actual points at (1,1).

and

*Assumption 2:* All grid points that fall on a lattice point of subclone A (circled in Figure 3d), have normal integer CNs in all other subclones than A.

This assumption means that no grid points at the lattice points are due to CN variation in other subclones, like the top red point in Figure 3a, or points of a second subclone with identical size to A as in Figure 3c.

If in addition to assumptions 1 and 2, we have property 2 holding, then condition 2 is satisfied, and we can estimate integer CNs of subclone A in the genome segments which fall at lattice points of subclone A. We will call these segments type A segments with respect to subclone A.

Further subclones may be identified using the same strategy. The point (1,1) will be part of the lattice points for each grid caused by CN alteration in one subclone when the integer CNs of the other subclones are normal. Therefore, (1,1) may be regarded as an observed grid point in search of points fulfilling property 1, even if there is no observed point there. With real data, the position of (1,1) will not be identified until a first subclone like A is found, so only subsequent subclone identifications can make use of it.

Even other subclones may be quantified under additional assumptions, as exemplified next.

### Example of further subclonal cell fraction and integer CN estimation

Imagine a tumor sample with an identified subclone A according to properties 1 and 2 and assumptions 1 and 2, and with

*Property 3:* The grid plot has at least one point below the lattice points of subclone A. (This indicates another subclone, with a larger cell fraction than A.)

For an example, see the green grid points of Figure 3d. Under the following assumption (which could be varied in different ways), condition 1 holds, so we can correctly identify a subclone (say C) in the tumor sample by its cell fraction.

*Assumption 3:* The horizontal distance between (1,1) and the average minor average CN of points below the lattice points of subclone A corresponds to a difference of integer CNs in subclone C of one.

We call segments with grid points falling below the lattice points of subclone A type C segments. If we further assume

*Assumption 4:* All type C segments have integer CNs (0,1) in subclone C.

then we could continue to deduce integer CNs in subclone A for those type C segments with grid points on a new set of lattice points, based on assumptions parallel to assumption 2 above.

### Identical array CNs can originate from different integer CNs

Given the unbiased, noise-free array CNs of Figure 4, it is unknown which of the lattice points (*g*_2_, *g*_2_), (*g*_3_, *g*_3_) or (*g*_4_, *g*_4_) corresponds to (1,1) integer copies. The scenarios 2, 3 and 4 involve different fractions *α* of cells displaying the colored grid point CN alterations, different sets of integer CNs, and different scaling factors between array CNs and average CNs. The following algebra shows how two consecutive scenarios (2 and 3) can result in identical total (that is, minor + major) array CNs (TCNs) and BAFs, and hence identical array CNs.

For scenario 3, let *c*_13_ and *c*_23_ denote the integer CNs in the aneuploid fraction *α*_3_ of cells of an arbitrary genome segment. Scenario 3 implies *α*_3_ = (*g*_3_ − *g*_2_)/*g*_3_ (Equation 2) and a scale factor *f*_3_ = *g*_3_ relating array CNs to average CNs. Hence the segment has:

$$ TC{N}_3=\left\{{\alpha}_3\left({c}_{13}+{c}_{23}\right)+2\left(1-{\alpha}_3\right)\right\}{g}_3 $$

$$ BA{F}_3^{upper}=\frac{\alpha_3{c}_{23}+\left(1-{\alpha}_3\right)}{\alpha_3\left({c}_{13}+{c}_{23}\right)+2\left(1-{\alpha}_3\right)}. $$

Next consider scenario 2, for which *α*_2_ = (*g*_2_ − *g*_1_)/*g*_2_, *f*_2_ = *g*_2_ and its integer CNs would be *c*_13_ + 1 and *c*_23_ + 1 for the same segment. We note that (*g*_2_ − *g*_1_) = (*g*_3_ − *g*_2_) = *α*_2_*g*_2_ = *α*_3_*g*_3_. Consequently

$$ \begin{array}{l}TC{N}_2=\left\{{\alpha}_2\left({c}_{13}+{c}_{23}+2\right)+2\left(1-{\alpha}_2\right)\right\}{g}_2\\ {}\kern4.2em =\left\{\frac{\alpha_3{g}_3}{g_2}\left({c}_{13}+{c}_{23}+2\right)+2\frac{g_2-{\alpha}_3{g}_3}{g_2}\right\}{g}_2\\ {}\kern4.2em ={\alpha}_3{g}_3\left({c}_{13}+{c}_{23}+2\right)+2\left({g}_2-{\alpha}_3{g}_3\right)={\alpha}_3{g}_3\left({c}_{13}+{c}_{23}\right)+2{g}_2\\ {}\kern4.2em ={\alpha}_3{g}_3\left({c}_{13}+{c}_{23}\right)+2\left({g}_3-{\alpha}_3{g}_3\right)\\ {}\kern4.2em =\left\{{\alpha}_3\left({c}_{13}+{c}_{23}\right)+2\left(1-{\alpha}_3\right)\right\}{g}_3=TC{N}_3\end{array} $$

$$ \begin{array}{l} BA{F}_2^{upper}=\frac{\alpha_2\left({c}_{23}+1\right)+\left(1-{\alpha}_2\right)}{\alpha_2\left({c}_{13}+{c}_{23}+2\right)+2\left(1-{\alpha}_2\right)}=\frac{\frac{\alpha_3}{\alpha_2}\left\{{\alpha}_2\left({c}_{23}+1\right)+\left(1-{\alpha}_2\right)\right\}}{\frac{\alpha_3}{\alpha_2}\left\{{\alpha}_2\left({c}_{13}+{c}_{23}+2\right)+2\left(1-{\alpha}_2\right)\right\}}=\\ {}\frac{\alpha_3{c}_{23}+{\alpha}_3+\left(\frac{\alpha_3}{\alpha_2}-{\alpha}_3\right)}{\alpha_3\left({c}_{13}+{c}_{23}\right)+2{\alpha}_3+2\left(\frac{\alpha_3}{\alpha_2}-{\alpha}_3\right)}=\frac{\alpha_3{c}_{23}+\frac{\alpha_3}{\alpha_2}}{\alpha_3\left({c}_{13}+{c}_{23}\right)+2\frac{\alpha_3}{\alpha_2}}=\frac{\alpha_3{c}_{23}+\frac{g_2}{g_3}}{\alpha_3\left({c}_{13}+{c}_{23}\right)+2\frac{g_2}{g_3}}=\frac{\alpha_3{c}_{23}+\frac{g_3-{\alpha}_2{g}_2}{g_3}}{\alpha_3\left({c}_{13}+{c}_{23}\right)+2\frac{g_3-{\alpha}_2{g}_2}{g_3}}=\\ {}\frac{\alpha_3{c}_{23}+\left(1-{\alpha}_3\right)}{\alpha_3\left({c}_{13}+{c}_{23}\right)+2\left(1-{\alpha}_3\right)}= BA{F}_3^{upper}\end{array} $$

### Resolving array CN scaling by approximate ploidy calculation

Given the position of (1,1) integer copies in a grid plot of noise-free array CNs, subclonal cell fractions and integer CNs can be derived for some segments and subclones in fortunate samples under certain assumptions. Unfortunately, the scaling (the position of (1,1)) of array CNs is generally unknown. By calculating subclone specific ploidies for each potential position of (1,1), FACS plots can sometimes help us resolve the scaling issue (Figure 5).

This is how we estimate the ploidy of a selected subclone A under properties 1 and 2 and assumptions 1 and 2. Given the potential scale factor *g*_*i*_ =1, subclonal integer CNs of A can be estimated for each segment *j* on lattice points of subclone A’s grid plot by ${\hat{c}}_{1j}=\left({a}_{1j}-{g}_{i-1}\right)/\left({g}_i-{g}_{i-1}\right)$, ${\hat{c}}_{2j}=\left({a}_{2j}-{g}_{i-1}\right)/\left({g}_i-{g}_{i-1}\right)$, where ${\overset{\rightharpoonup }{a}}_j=\left({a}_{1j},{a}_{2j}\right)$ are minor and major array CNs.

If

*Property 4:* The fraction of the genome which cannot be resolved for integer CNs in subclone A is negligible with respect to the subclone’s average CN.

then we can estimate the ploidy of subclone A by summing up these estimated integer CNs to ${\displaystyle \sum_j}\left[{l}_j\left({\hat{c}}_{1j}+{\hat{c}}_{2j}\right)\right]$, where *l*_*j*_ is the genomic length of segment *j*, and dividing the result by ${\displaystyle \sum_j}{l}_j$.

If at least one of the following holds, approximate overall ploidy estimates (across all the tumor cells) can be calculated from the array CNs for each potential position of (1,1), and an independent overall ploidy estimate from, for example, FACS runs may help resolve the array CN scaling.

*Property 5:* The fraction of the genome which cannot be resolved for integer CNs (via subclones) is negligible with respect to the sample’s average CN.

*Assumption 5:* The average CN across the part of the genome which can be assessed for integer CNs (via subclones) is similar to the average CN across the rest of the genome.

This is how, under either property 5 or assumption 5, we estimate overall ploidy in a sample with one evident subclone A. Given the potential scale factor *g*_*i*_ =1, the subclone A cell fraction is *α* = (*g*_*i*_ − *g*_*i* −1_)/*g*_*i*_. Let:

$$ overall\; ploidy=\left(\frac{\alpha }{p}\right){\displaystyle \sum_j}\left[{\pi}_i\left({\hat{c}}_{1j}+{\hat{c}}_{2j}\right)\right]+2\left(\frac{1-\alpha }{p}\right) $$

where *π*_*j*_ is the fraction of the genome associated with segment *j* and *p* is a pathologist’s estimate of sample purity. The relative size of subclone A among the tumor cells, *α*/*p*, is also known as the subclone’s ccf*.*

In samples with one evident subclone A as well as evidence of a larger subclone, C, we may refine the overall ploidy estimate with the integer CN estimates mentioned earlier.

### VAFs can sometimes help deduce the scaling of array CNs

In this section we use the example in Figure 4 to explain the use of mutation VAFs to deduce the scaling of array CNs. This procedure again requires a set of subjectively chosen assumptions and only works under fortunate circumstances.

A grid plot of simulated, noise- and bias-free array CNs is shown in Figure 4a. The scale of the array CNs is unknown. The four colored points suggest the grid pattern drawn for a subclone A, but it is unknown whether (1,1) integer copies happen at (*g*_2_, *g*_2_), (*g*_3_, *g*_3_) or (*g*_4_, *g*_4_). The three scenarios areis illustrated in Figure b-d, which all have one colored column for each of the colored genome segments in Figure 4a. Equally between the panels, two of the segments have mutations on them (red crosses), with VAFs as shown on the y-axes (simulated without noise or bias).

Each different scaling suggests different integer CNs (*c*_1_, *c*_2_) for the segments (labels on x-axes). For example, if *g*_2_ = 1 (Figure 4b), the blue segment must have integer CNs (2,2) in subclone A. Given a pair of integer CNs (*c*_1_, *c*_2_), expected VAF levels can be derived under certain assumptions.

If

*Property 6:* A mutation sits on a segment that falls on a lattice point of a subclone A.

and we assume

*Assumption 6:* Mutations sit on a number of the *c*_1_ + *c*_2_ local chromosomal copies in subclone A cells only.

in addition to relying on properties and assumptions 1 to 2, then we would expect VAF levels only in {*αc*/*D*, *c* =1, 2, …, *c*_1_ + *c*_2_}, where *α* is the cell fraction of subclone A, and *D* is the total (minor + major) average CN at the mutation’s genomic position, *D* = *α*(*c*_1_ + *c*_2_) +2(1 − *α*). Under these circumstances, and if *g*_2_ = 1 (Figure 4b), the mutation on the blue segment in the example would sit on 1, 2, 3 or 4 of the 2 + 2 chromosomal copies. The four corresponding expected VAF levels, simulated with sample purity 90%, have been drawn as continuous, horizontal lines. The other scenarios, *g*_3_ = 1 (Figure 4c) and *g*_4_ = 1 (Figure 4d), suggest other VAF levels (continuous, horizontal lines).

If the sample purity *p* is known (for example, from a pathologist’s examination) and if, instead of assumption 6, we assume

*Assumption 7:* Mutations sit on one or both of the chromosomal copies of all tumor cells other than subclone A.

then we would expect VAF levels only in {(*p* − *α*)*c*/*D*, *c* =1, 2}. For mutations present in both subclone A cells and all other tumor cells, assume

*Assumption 8:* Mutations sit on a number of the *c*_1_ + *c*_2_ local chromosomal copies in subclone A cells, and on one or both of the chromosomal copies of all tumor cells other than subclone A.

We call such mutations clonal, and for these we expect VAF levels only in {(*αc* + (*p* − *α*)*d*)/*D*, *c* =1, 2, …, *c*_1_ + *c*_2_, *d* =1, 2}. If *g*_2_ = 1 (Figure 4b), assumptions 7 and 8 and a purity of 90% give the 10 expected VAF levels drawn as dashed horizontal lines for the blue segment.

Pretending that assumptions 6 to 8 cover all possible locations of mutations on segments that fall on the grid plot lattice points fulfilling conditions 1 and 2, scenario *g*_4_ = 1 (Figure 4d) can be ruled out - one mutation VAF in inexplicable as it does not coincide with a horizontal line. If we make the assumption that

*Assumption 9:* Mutations are heterozygous and present on all the copies of its homologue (thick continuous or dashed lines).

we can also rule out the scenario *g*_2_ = 1 (Figure 4b) and fix the average CNs of Figure 4c for this sample. Some samples have mutations which can help resolve the array CN scaling, like this. Other samples may have too few mutations, even in this optimal world with no noise in VAFs or segmented CN data.

### SNP array preprocessing and segmentation

Genome-wide SNP analysis of tumor and matched normal samples was performed at AROS Applied Biotechnologies a/s (Aarhus, Denmark) on Affymetrix Genome-Wide Human SNP Arrays 6.0 (Affymetrix, Santa Clara, CA, USA) following the manufacturer’s instructions, with the 52 tumor samples and the 29 available matched normal samples. The arrays were preprocessed with the ASCRMAv2 single-array method in the aroma.affymetrix R package [30,31], and further adjusted for SNP-specific allelic crosstalk with CalMaTe [32]. Total (signal A plus signal B) tumor SNP array signals were normalized (divided by) towards total SNP array signals of matched normal samples where available, or otherwise position-specific median total SNP array signals across the normal samples, giving TCNs for all tumors. BAFs were obtained and processed using TumorBoost. Allele-specific CN segments were identified from TCNs and BAFs with the paired or non-paired PSCBS method [33] for samples with or without a matched normal sample. After this step we have a minor and a major array CN for each segment, equal to the median TCN(1 - BAF_upper_) and TCN(BAF_upper_) across the SNPs in the segment. Two arrays failed this preprocessing.

The segmented array CNs were refined with HAPSEG [34], which phases the SNP alleles by comparing the sample-specific SNP data to large databases of normal sample SNP datasets. We let HAPSEG join up the adjacent segments we supplied with similar CNs to a limited extent (seg.merge.thresh =1 or 10^-10^ for different samples). HAPSEG significantly reduced CN bias in segments with allelic balance, which originally occurred because segment BAFs were estimated by the median distance between individual SNP BAF levels and 0.5, which is >0 even for segments with allelic balance. It also rescales the segment CNs so that they average to 1 for single homologues. The resulting homologue-specific segment CNs are referred to as array CNs throughout this paper.

All data analyses in this study were made with R [35] unless otherwise stated.

### WES variant detection

DNA was extracted using the DNeasy Blood and Tissue Kit® (Qiagen, Venlo, Netherlands) following the manufacturer’s instructions. DNA concentration was measured using the NanoDrop 1000 instrument (Thermo Scientific, Waltham, MA, USA). Whole exome sequencing was performed at DNAVision (Gosselies, Belgium). Genomic libraries from the tumor and matched normal samples were generated using the SureSelectXT Reagent Kit HSQ (Agilent Technologies, Santa Clara, CA, USA) following the manufacturer’s instructions. Enrichment was performed using the SureSelectXT Human All Exon V4 + UTRs kit (Agilent) following the manufacturer’s instructions.

Exome read alignment, filtering, variant calling and annotation were performed as follows. Cutadapt 1.1 [36] was used for quality-based adaptor trimming, sequence reads were aligned to the GRCh37/hg19 human reference genome using bwa-aln 0.7.7-r441 [37] and duplicate reads marked using Picard tools [38]. Aligned reads for each tumor-normal sample pair were combined into one alignment file in BAM format, followed by local indel realignment and base quality recalibration using the Genome Analysis Tool Kit (GATK) software [39]. The MuTect 2.7-1-g42d771f [40] program was used to identify somatic point mutations. Predictions not labeled as ŒREJECT^1^ were accepted as confident somatic mutation predictions and considered for subsequent downstream validation and analysis steps. Variant annotation was performed using the Oncotator web-based service [41]. VAFs denote the number of reads with the detected variant as a fraction of all reads at the corresponding genomic position.

### Scaling bias in SNP array B allele fractions

Grid plot skewness (Figure 7) is adjusted for as follows.

We assume that an unknown fraction *α* of the (germline-contaminated) tumor sample contributes the most visible, regular CN grid in the plot, and refer to this as our main subclone A. Note that subclone A may be all the tumor cells in the sample, in which case *α* is the sample purity, or it may be a true subclone of tumor cells. Within subclone A, each true single homologue average CN *e* should, theoretically, follow:

3 $$ e\in \left\{\left(1-\alpha \right)+\alpha c,\;c=0,\;1,\;2, \dots \right\}, $$

where *c* refers to the integer CNs in subclone A. We model the observed minor and major array CNs $\overset{\rightharpoonup }{a}=\left({a}_1,{a}_2\right)$ of an arbitrary CN segment as if they have been subject to a plane rotation $$ F=\left(\begin{array}{cc}\hfill {f}_{11}\hfill & \hfill {f}_{12}\hfill \\ {}\hfill {f}_{21}\hfill & \hfill {f}_{22}\hfill \end{array}\right) $$ of the average CNs $\overset{\rightharpoonup }{e}=\left({e}_1,{e}_2\right)$, which are functions of the integer CNs $\overset{\rightharpoonup }{c}=\left({c}_1,{c}_2\right)$ in A: $\overset{\rightharpoonup }{a}=F\overset{\rightharpoonup }{e}$, that is:

$$ \left(\begin{array}{c}\hfill {a}_1\hfill \\ {}\hfill {a}_2\hfill \end{array}\right)=\left(\begin{array}{cc}\hfill {f}_{11}\hfill & \hfill {f}_{12}\hfill \\ {}\hfill {f}_{21}\hfill & \hfill {f}_{22}\hfill \end{array}\right)\left(\begin{array}{c}\hfill 1-\alpha +\alpha {c}_1\hfill \\ {}\hfill 1-\alpha +\alpha {c}_2\hfill \end{array}\right). $$

Our aim is to estimate the rotation matrix *F*, assumed to be common to all the CN segments in the sample, and hence to derive the skewness adjusted array CNs ${\overset{\rightharpoonup }{a}}^{\hbox{'}}={\hat{F}}^{-1}\overset{\rightharpoonup }{a}$. The matrix *F* involves a scale factor dependent on the unknown fraction *α*. For the sake of grid rotation we use the maximum *α* that fits the array CNs. This is the scaling scenario which corresponds to the smallest possible position of (1,1). (The final scaling step for estimation of *α* and the integer CNs {(*c*_1_, *c*_2_)} is based on observed VAFs as described below.)

We find M-estimates of *α* and the matrix *F* numerically [42,43] by minimizing of the sum of the distances from each CN segment $\overset{\rightharpoonup }{a}$ to its closest skewed lattice point $F\overset{\rightharpoonup }{e}$ weighted by the robust Tukey function and the length of the genome segment corresponding to each $\overset{\rightharpoonup }{a}$. An M-estimator [44,45] is a generalization of the maximum likelihood (ML-) estimator. It minimizes the summed values of a function *ρ*, $\hat{F}= argmi{n}_F{\displaystyle \sum_{i=1}^n}\rho \left({r}_i\right)$, where *ρ* is similar to but not necessarily a likelihood function. We let ${r}_i={w}_i\left|{\overset{\rightharpoonup }{a}}_i-F{\overset{\rightharpoonup }{e}}_i\right|$ for each segment *i*, where $F{\overset{\rightharpoonup }{e}}_i$ is the closest lattice point to ${\overset{\rightharpoonup }{a}}_i$, and *w*_*i*_ is a weight ≤1 determined by the length *l*_*i*_ of segment *i* (typically ${w}_i=1-{e}^{-{l}_i/500000}$ ) to downweight short segments (typically <1 Mb), which might have less reliable array CNs. Tukey’s *ρ* function truncates its input in a smooth fashion, so that observations far away have a limited influence on our estimate. In this way, we avoid segments that violate the grid (for example, because they belong to a different subclone), blurring our estimate of *F*. Figure 7c shows a resulting grid plot after grid rotation.

Starting values *α*_0_ and *F*_0_ for the grid rotation are derived in two steps. Step 1 estimates a pre-start matrix $$ {F}^{\prime }=\left(\begin{array}{cc}\hfill {f}_{11}^{\hbox{'}}\hfill & \hfill {f}_{12}^{\hbox{'}}\hfill \\ {}\hfill {f}_{21}^{\hbox{'}}\hfill & \hfill {f}_{22}^{\hbox{'}}\hfill \end{array}\right) $$, with *F*′ = *αF* so that a pre-rotation ${\left({F}^{\prime}\right)}^{-1}\overset{\rightharpoonup }{a}$ gives a non-skewed grid plot with unit increments between vertical and horizontal lattice points (Figure 7b):

$$ \left(\begin{array}{c}\hfill {a}_1\hfill \\ {}\hfill {a}_2\hfill \end{array}\right)=\frac{1}{\alpha }{F}^{\prime}\left(\begin{array}{c}\hfill 1-\alpha +\alpha {c}_1\hfill \\ {}\hfill 1-\alpha +\alpha {c}_2\hfill \end{array}\right) $$

$$ ={F}^{\prime}\left(\begin{array}{c}\hfill \frac{1-\alpha }{\alpha }+{c}_1\hfill \\ {}\hfill \frac{1-\alpha }{\alpha }+{c}_2\hfill \end{array}\right), $$

$$ {\left({F}^{\prime}\right)}^{-1}\left(\begin{array}{c}\hfill {a}_1\hfill \\ {}\hfill {a}_2\hfill \end{array}\right)=\left(\begin{array}{c}\hfill \frac{1-\alpha }{\alpha }+{c}_1\hfill \\ {}\hfill \frac{1-\alpha }{\alpha }+{c}_2\hfill \end{array}\right). $$

Rough settings are collected by manually selecting informative clusters in the skewed grid plot (Figure 7a):

- *d*_*y*_ = *vertical component of the distance between two* ‘*vertically*’ *consecutive clusters*
- *d*_*x*_ = *horizontal component of the distance between two* ‘*horizontally*’ *consecutive clusters*
- *slope*_*y*_ = slope of the line through two ‘*vertically*’ *consecutive clusters*
- *slope*_*x*_ = slope of the line through two ‘*horizontally*’ *consecutive clusters*

Let (*c*_1*j*_, *c*_2*j*_) and (*c*_1*j* +1_, *c*_2*j* +1_) be the (unknown) integer CNs of subclone A seen as two consecutive, ‘vertical’ clusters in the skewed grid plot, such that *c*_1*j*_ +1 = *c*_1*j* +1_ and *c*_2*j*_ = *c*_2*j* +1_. Then

$$ {d}_y=\left({f}_{11}^{\hbox{'}}\left[\frac{1-\alpha }{\alpha }+{c}_{1j+1}\right]+{f}_{12}^{\hbox{'}}\left[\frac{1-\alpha }{\alpha }+{c}_{2j+1}\right]\right)-\left({f}_{11}^{\hbox{'}}\left[\frac{1-\alpha }{\alpha }+{c}_{1j}\right]+{f}_{12}^{\hbox{'}}\left[\frac{1-\alpha }{\alpha }+{c}_{2j}\right]\right)={f}_{11}^{\hbox{'}} $$

and similarly ${d}_x={f}_{22}^{\hbox{'}}$, $slop{e}_y={f}_{11}^{\hbox{'}}/{f}_{21}^{\hbox{'}}$ and $slop{e}_x={f}_{12}^{\hbox{'}}/{f}_{22}^{\hbox{'}}$. We estimate the pre-start matrix *F*′ by:

$$ \left\{\begin{array}{c}\hfill {f}_{11}^{\hbox{'}}={d}_y\hfill \\ {}\hfill {f}_{22}^{\hbox{'}}={d}_x\hfill \\ {}\hfill {f}_{21}^{\hbox{'}}={f}_{11}^{\hbox{'}}/ slop{e}_y\hfill \\ {}\hfill {f}_{12}^{\hbox{'}}={f}_{22}^{\hbox{'}} slop{e}_x.\hfill \end{array}\right. $$

In step 2 we estimate *α*_0_, the maximum possible fraction *α* such that 0 < *α* ≤1. Starting from a selected lattice point with allelic balance a grid with step size one is imposed on the pre-rotated Figure 7b, stretching as close to zero as possible with all gridlines positive. Let the array CN levels of the first two horizontal gridlines be *α*_*i*_ and *α*_*ii*_. Using Equation 3 we derive ${\alpha}_{max}^x=\left({a}_{ii}-{a}_i\right)/{a}_{ii}$, and similarly for ${\alpha}_{max}^y$ from vertical gridlines. We set start values for the numerical optimization to ${\alpha}_0=\left({\alpha}_{max}^x+{\alpha}_{max}^y\right)/2$ and *F*_0_ = *F*′/*α*_0_.

What is the origin of the grid bias, the skewness? We further investigate the SNP array components from which array CNs are computed: TCNs and BAFs. Note that the BAFs here refer to the upper BAF of each segment, which is always between 0.5 and 1.

**Figure 13:**
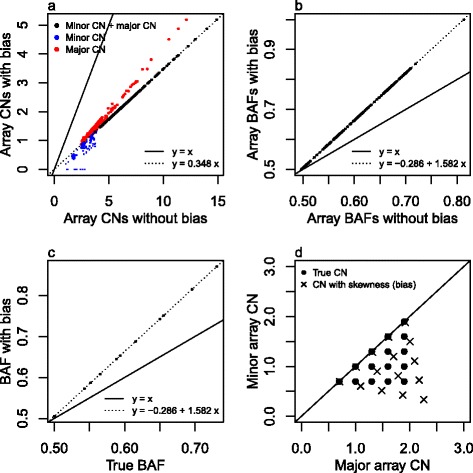
**Origin of skewness. (a)** Sample 45 before versus after rotation TCNs are exactly proportional, whereas minor or major CNs show a more complicated difference. **(b)** Sample 45 BAFs before rotation (upper) carry a 0.5 centered scaling bias compared to after rotation (upper), a bias which causes grid plot skewness. **(c)** Imposed BAF bias on simulated array data in (d). **(d)** Grid plots of true and BAF-induced biased array CNs in a simulated dataset.

We plot the observed total array CNs *a*_1_ + *a*_2_ towards the rotated, supposedly unbiased ${a}_1^{\hbox{'}}+{a}_2^{\hbox{'}}$ (Figure 13a) as well as observed (upper) BAFs *a*_2_/(*a*_1_ + *a*_2_) towards the rotated (upper) BAFs ${a}_2^{\hbox{'}}/\left({a}_1^{\hbox{'}}+{a}_2^{\hbox{'}}\right)$ (Figure 13b). Assuming the rotated CNs are truly proportional to the true average CNs, the graph suggests the original total CNs (since proportional to rotated CNs) are indeed also proportional to the true average CNs. The biased total array CNs and the rotated ones have different scale factors, but that cannot be the cause or adjustment for skewness. In Figure 13a single homologue (minor and major) original and rotated CNs are not just proportional to each other, but seem subject to bias related to single CN magnitude. Indeed, Figure 13b suggests BAFs carry scaling bias centered at 0.5. If had plotted the lower BAFs instead (which are between 0 and 0.5), Figure 13b would have shown points on the bottom left extension of the dotted line. Either way the deviation between BAFs before and after rotation is small for BAFs close to 0.5 and larger further away. To investigate whether such a BAF bias may cause grid plot skewness, we simulate a set of array CNs as in Figure 1, and derive its true BAFs. We then created a biased dataset with total array CNs as in Figure 1 but with BAFs biased (Figure 13c) according to the estimated linear model in the real dataset (Figure 13b). Plotting both the true and the biased simulated array CNs in Figure 13d reveals that a 0.5 centered scaling bias of BAFs may indeed cause skewness in grid plots.

The connection between grid plot skewness and bias in BAFs introduces a relationship between the expected association in Figure 13, *BAF*_*observed*_ = *k* + *l* × *BAF*_*true*_, and the rotation matrix *F* up to a scaling constant *C*:

4 $$ F=C\left(\begin{array}{cc}\hfill 1-k\hfill & \hfill 1-k-l\hfill \\ {}\hfill k\hfill & \hfill k+l\hfill \end{array}\right). $$

For the example sample 45 we estimated *BAF*_*observed*_ = −0.286 + 1.582*BAF*_*rotated*_, so we should have *F* proportional to $$ \left(\begin{array}{cc}\hfill 1.286\hfill & \hfill -0.286\hfill \\ {}\hfill -0.296\hfill & \hfill 1.296\hfill \end{array}\right) $$, and from the grid rotation we indeed estimated:

$$ F=\left(\begin{array}{cc}\hfill 0.447\hfill & \hfill -0.103\hfill \\ {}\hfill -0.101\hfill & \hfill 0.452\hfill \end{array}\right)=0.349\left(\begin{array}{cc}\hfill 1.280\hfill & \hfill -0.294\hfill \\ {}\hfill -0.289\hfill & \hfill 1.295\hfill \end{array}\right). $$

Equation 4 imposes the restriction *f*_11_ + *f*_21_ = *f*_12_ + *f*_22_ on *F*, which can sometimes help the numerical optimization.

The BAF bias causes segments with very low minor CNs to get upper BAFs biased down to 1 in the array preprocessing steps. Such segments will appear as a horizontal bottom line in original grid plots, and a sloped bottom line after grid rotation. We believe that these segments should have had a constant minor array CN, and so we project the corresponding grid plot points vertically down to the observed bottom line of constant minor array CNs when the latter is evident.

Unless otherwise stated, we refer to array CNs as rotated array CNs after projection of bottom sloped line array CNs, and we drop the prime from ${\overset{\rightharpoonup }{a}}^{\hbox{'}}$.

### Estimation of subclonal architecture, cell fractions and integer CNs in RESPONSIFY samples

The cell fraction and integer CNs are estimated for type A segments of cells in the most evident subclone A. According to our assumptions all other cells are diploid in these segments.

CN alteration in type B segments may be due to further CN alteration in daughter subclones of A or by CN alteration in subclones independent of A. For some segments which have many mutations we can deduce the subclonal origin via the mutation VAFs as in the following example. In Figure 11, VAFs in type A segments (blue) match the expected VAF levels of subclone A ({*αc*/*D*, *c* = *c*_1_, *c*_2_} with notation as above but now referring to estimates from real data), which is reassuring for our analyses. VAFs in type B segments (pink) also match expected VAF levels of subclone A ({*αc*/*D*, *c* = *c*_1_, *c*_2_} with *c*_1_, *c*_2_ being fractional rather than integer CNs). This suggests the true CNs indeed meet the fractional *c*_1_, *c*_2_ in a fraction *α* of the sample cells, which in turn suggests subclone A has daughter subclones with further CN alteration in these segments.

For 25 of the 48 samples resolved for array scaling we identified an additional subclone C by the existence of a lower grid pattern in the grid plot (see Materials and methods). In these cases we call the lower grid pattern segments type C segments, estimate the subclone C cell fraction approximately, assign integer CNs (0,1) to the subclone C type C segments, and estimate integer CNs of subclone A in the type C segments via Equation 2.

All our samples lack regular grid patterns in high array CN segments (red in Figure 8). We call these type D segments. Their CN alteration may take place in subclone A (but the grid is not regular because the proportionality between array CNs and average CNs breaks down with high SNP array intensities), in subclone C or in any other subclone. As for type B segments, some segments which have many mutations can be assigned to a subclone via the mutation VAFs. In the example of Figure 11, the VAFs in type D segments (red) match the expected VAF level of the minor homologue of subclone A ({*αc*_1_/*D*} where *c*_1_ is a fractional CN. This suggests that any CN alteration in the minor homologue takes place in subclone A.

### Clonal or subclonal mutations in RESPONSIFY samples

Continued from the ‘Clonal or subclonal mutations’ section in Results. Segment types are exemplified in Figure 8.

For samples with only one identified subclone A, we classify each type A mutation as clonal if the CI falls above (*αc*_1_ + (*p* − *α*))/*D* − *δ*, with *δ* =0.1 (non-inferiority test at significance level 5%), *α*, *c*_1_ and *c*_2_ estimates for subclone A and *D* the estimate of the local minor + major average CN. We classify a mutation as subclonal if its CI falls below (*αc*_1_ + (*p* − *α*))/*D* (one-sided inferiority test at significance level 5%). Some mutations will be called ambiguous. For subclonal mutations we further test whether they sit on subclone A or not, with equality tests significant if the CI falls within *αc*_1_/*D* ± *δ* or *αc*_2_/*D* ± *δ*.

If, in addition to a most evident subclone A, a sample has another identified subclone C in a fraction *γ* of the cells, we assess whether the subclonal type A mutations seem to sit on A only (CI ∈ *αc*_1_/*D* ± *δ* or CI ∈ *αc*_2_/*D* ± *δ*), on C only (CI ∈ *γ*/*D* ± *δ*) or on both A and C (CI ∈ (*αc*_1_ + *γ*)/*D* ± *δ* or CI ∈ (*αc*_2_ + *γ*)/*D* ± *δ*). The extended procedure creates more ambiguous mutations, since we only allow non-ambiguous classifications.

Mutations of type B or D are classified like A mutations (so that equivalence to a specific subclonal expected VAF level suggests further daughter subclones with CN alteration in B segments) unless the sample has an identified subclone C. In case of the latter, segments of type B or D may have (I) integer CNs (*c*_1*a*_, *c*_2*a*_) in subclone A and (1, 1) in other tumor cells, or, they may have (II) integer CNs (*c*_1*c*_, *c*_2*c*_) in subclone C and (1, 1) in other tumor cells, or something else. We classify the type B and D mutations as outlined for type A mutations (based on I) in parallel with an analogous procedure based on II. Only mutations for which both classifications agree are finally assigned a class different from ambiguous.

Type C mutations are classified as type B or D ones, except in samples with an identified subclone C, for which we set the minor homologue integer CNs in subclone C to (0, 1) instead of (1, 1), and adjust the subclone A integer CNs according to Equation 2.

Approximately 35% of the 52 RESPONSIFY samples have the majority of type B and D VAFs matching expected VAF levels for CN alteration in subclone A. Most samples have no or only a handful of mutations in type C segments.

### Grid plots reveal bias

This section illustrates the benefit of two-dimensional grid plots compared with one-dimensional histograms, in order to reveal bias in array CNs.

**Figure 14:**
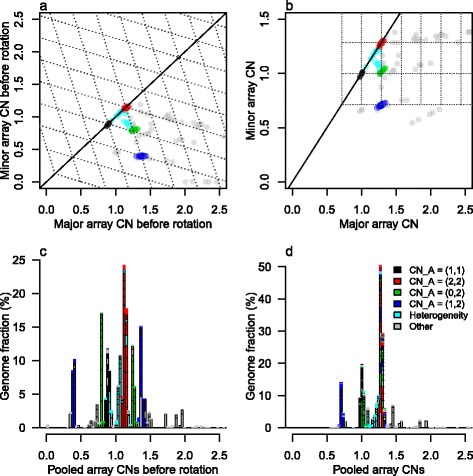
**Sample 11 grid plots and Absolute histograms. (a)** Original grid plot. **(b)** Grid plot after rotation. **(c)** Absolute histogram based on original array CNs. **(d)** Absolute histogram based on rotated array CNs. Note that all plots show the array CNs scaled to equal average CNs, so that the level one (1) corresponds to normal, single haplotype CNs.

Each point in the original (Figure 14a) and rotated (Figure 14b) array CN grid plots shows the minor and major array CN of a genome segment. In the Absolute software, the minor and major array CNs are pooled and shown in (one-dimensional) histograms (Figure 14c for original and Figure 14d for rotated array CNs) with heights proportional to segment lengths. Hence, each segment is represented twice in the histograms - once with the minor array CN and once with the major. Segments with subclone A two-dimensional CN estimates (1,1), (2,2), (0, 2) and (1,2) have been colored equally in all four panels (black, red, blue, green). The cyan segments have non-integer CNs (between 1 and 2) with respect to subclone A. They may have further heterogeneity within subclone A or originating from a subclone independent of A. Absolute searches for equally interspaced peak centers in the histogram with a maximum likelihood algorithm, and each peak is assigned an integer CN estimate. Segments that fall significantly far from their closest peak centers are classified as subclonal, under the assumptions that (i) only one pattern of equally interspaced peaks can occur, and (ii) the pattern reflects the clonal CNs of all tumor cells in the sample. According to the CN coloring in Figure 14, three colored histogram peaks are expected: CN =0 (blue), CN =1 (black, green) and CN =2 (red, blue, green). In the histogram of original array CNs (Figure 14a) it is hardly possibly to identify the three CN levels, their centers are not equally interspaced, and non-integer CNs (cyan) are intermixed with the integer CNs. Since all our samples have skewness, Absolute did not assign integer CNs optimally.

### Data and implementation

The preprocessed array CN data for the six samples discussed in this paper are available as Additional files 1, 2, 3, 4, 5 and 6, while the Oncotator annotated Mutect variants for two of these samples are available in Additional files 7 and 8. A CRAN package to identify grid patterns, perform our grid rotation algorithm and calculate the ITH endpoint will be available shortly with full documentation under the name ‘Gridith’. A “Gridith” beta version is available at https://github.com/fcaramia/GRIDITH.

## Additional files

Additional file 1
**Sample 5 preprocessed segment array CNs.**
Additional file 2
**Sample 9 preprocessed segment array CNs.**
Additional file 3
**Sample 11 preprocessed segment array CNs.**
Additional file 4
**Sample 16 preprocessed segment array CNs.**
Additional file 5
**Sample 29 preprocessed segment array CNs.**
Additional file 6
**Sample 45 preprocessed segment array CNs.**
Additional file 7
**Sample 5 mutect variants oncotator annotated mindepth10.**
Additional file 8
**Sample 16 mutect variants oncotator annotated mindepth10.**

## Abbreviations

BAF: B allele fraction
ccf: cancer cell fraction
CI: confidence interval
CN: copy number
FACS: fluorescence-activated cell sorting
ITH: intra-tumor heterogeneity
LOH: loss of heterozygosity
SNP: single nucleotide polymorphism
TCN: total copy number
VAF: variant allele fraction
WES: whole exome sequencing
WGS: whole genome sequencing
